# Ocular biomarkers for cognitive impairment in nonagenarians; a prospective cross-sectional study

**DOI:** 10.1186/s12877-020-01556-1

**Published:** 2020-04-28

**Authors:** Jacoba A. van de Kreeke, Nienke Legdeur, Maryam Badissi, H. Ton Nguyen, Elles Konijnenberg, Jori Tomassen, Mara ten Kate, Anouk den Braber, Andrea B. Maier, H. Stevie Tan, Frank D. Verbraak, Pieter Jelle Visser

**Affiliations:** 1 Ophthalmology Department, Amsterdam UMC, location VUmc, De Boelelaan 1117, 1081HV Amsterdam, The Netherlands; 2 Alzheimer Center Amsterdam, Department of Neurology, Amsterdam Neuroscience, Vrije Universiteit Amsterdam, Amsterdam UMC, Amsterdam, The Netherlands; 3 Department of Biological Psychology, VU University Amsterdam, Amsterdam, The Netherlands; 4 Department of Medicine and Aged Care, @AgeMelbourne, Royal Melbourne Hospital, University of Melbourne, Melbourne, Australia; 5 Department of Human Movement Sciences, @AgeAmsterdam, Amsterdam Movement Sciences, Vrije Universiteit Amsterdam, Amsterdam, The Netherlands

**Keywords:** Retina, Optical coherence tomography, Fundus photography, Singapore I vessel assessment, Nonagenarians, Ocular biomarkers, Cognitive impairment

## Abstract

**Background:**

Ocular imaging receives much attention as a source of potential biomarkers for dementia. In the present study, we analyze these ocular biomarkers in cognitively impaired and healthy participants in a population aged over 90 years (= nonagenarian), and elucidate the effects of age on these biomarkers.

**Methods:**

For this prospective cross-sectional study, we included individuals from the EMIF-AD 90+ study, consisting of a cognitively healthy (*N* = 67) and cognitively impaired group (*N* = 33), and the EMIF-AD PreclinAD study, consisting of cognitively healthy controls aged ≥60 (*N* = 198). Participants underwent Optical Coherence Tomography (OCT) and fundus photography of both eyes. OCT was used to asses total and individual inner retinal layer thickness in the macular region (Early Treatment Diabetic Retinopathy Study circles) as well as peripapillary retinal nerve fiber layer thickness, fundus images were analyzed with Singapore I Vessel Assessment to obtain 7 retinal vascular parameters. Values for both eyes were averaged. Differences in ocular biomarkers between the 2 nonagenarian groups were analyzed using linear regression, differences between the individual nonagenarian groups and controls were analyzed using generalized estimating equations.

**Results:**

Ocular biomarkers did not differ between the healthy and cognitively impaired nonagenarian groups. 19 out of 22 ocular biomarkers assessed in this study differed between either nonagenarian group and the younger controls.

**Conclusion:**

The ocular biomarkers assessed in this study were not associated with cognitive impairment in nonagenarians, making their use as a screening tool for dementing disorders in this group limited. However, ocular biomarkers were significantly associated with chronological age, which were very similar to those ascribed to occur in Alzheimer’s Disease.

## Background

The eye, and more specifically the retina, shares many similarities with the brain. Both are derived from the same embryological tissue and consist of a complex combination of neuronal tissue and glial cells [1, 2]. One could consider the retina to be an extension of the brain [1, 2]. Assessment of the retina may provide information on diseases causing cognitive impairment, such as Alzheimer’s disease (AD). Therefore, studies have been performed on the diagnostic value of optical techniques used to examine the retina in AD, as these ocular markers may be less invasive and cheaper than most established biomarkers for brain diseases [3–8].

There is ample evidence of retinal changes in dementia [3–8]. In AD, retinal thinning has been observed on Optical Coherence Tomography (OCT), especially of the inner layers [3, 5]. Studies have also shown differences between individuals with and without dementia in the (micro)vascular state of their retinal vessels (such as vessel width, tortuosity and fractal dimension) through fundus photography, although these results were sometimes contradicting [8–11].

It is estimated that up to 40% of individuals aged 90 and over suffer from dementia and as such, this group is an important target group for potential new biomarkers for dementia [12]. In nonagenarians (individuals between the ages of 90–100), dementia is mostly caused by mixed pathologies, including AD and vascular brain damage [13, 14]. This may be reflected in the retinal biomarkers, causing the expected changes due to for example AD (retinal thinning and changes in microvasculature) to be less discriminative in this older population compared to younger individuals. Furthermore, many of the changes occurring in the retina of individuals with dementia also occur naturally with the aging process [15, 16]. These factors could mean that ocular biomarkers are much less suitable in a significantly aged group.

In this cross-sectional study, we aimed to 1) investigate whether retinal (layer) thickness and retinal vascular parameters differ between cognitively healthy and cognitively impaired nonagenarians and 2) elucidate the effects of age on these ocular biomarkers.

## Methods

### Participants

This study consists of combined data from 2 Amsterdam UMC sub-studies of the European Medical Information Framework for Alzheimer’s Disease (EMIF-AD): the EMIF-AD 90+ study and the EMIF-AD PreclinAD cohort. The 90+ study consists of cognitively healthy and cognitively impaired subjects, aged ≥90 or over. For extensive recruitment information we refer to our set-up paper of this study by Legdeur et al. [17]. The PreclinAD cohort [18] is a cohort consisting of cognitively healthy participants (monozygotic twins) aged ≥60, recruited from the Netherlands Twin Registry [19]. The studies adhered the Tenets of the Declaration of Helsinki and written informed consent was obtained from all participants. The Medical Ethics Committee of the Amsterdam UMC approved both studies.

For complete in- and exclusion criteria of the EMIF-AD 90+ study, we refer to our set-up paper of this study by Legdeur et al. [17]. In short, inclusion criteria for the cognitively normal group of the 90+ study were: age ≥ 90 years and cognitively healthy This group is referred to as ‘healthy nonagenarian’ group.

Inclusion criteria for individuals with cognitive impairment (CI) of the 90+ study were: a diagnosis of amnestic Mild Cognitive Impairment (aMCI) [20] or a diagnosis of probable or possible AD [21]. As during the study we had difficulties identifying subjects of 90 years and older with aMCI or probable or possible AD, we broadened the inclusion criteria in this group to subjects older than 85 years. Six individuals from this group were aged 85–90 years. This group is referred to as ‘CI nonagenarian’ group.

Inclusion criteria for the PreclinAD study were: age ≥ 60 years, monozygosity and cognitively healthy. For complete in−/exclusion criteria we refer to the set-up paper of this study by Konijnenberg et al. [18]. This group is referred to as the ‘control’ group.

From the total 298 participants included in the cohorts, 51 (17.1%) participants were excluded for both the OCT and SIVA analyses, but these were not necessarily the same participants, although there was a high overlap. For the OCT analyses, 9 were excluded due to low quality scans/failed imaging and 42 due to ophthalmological pathology. For the SIVA analyses, 24 were excluded due to low quality images/failed imaging and 27 due to ophthalmological pathology. Interfering ophthalmological pathology consisted mostly of glaucoma and (severe) AMD. Additional file 1 shows the reasons for exclusion in more detail, categorized per group (control, heathy nonagenarian and CI nonagenarian). Although the two study populations (i.e. for OCT and SIVA analyses) were slightly different from each other in terms of included individuals, they were very similar in their demographics, and statistical analyses revealed no significant differences. As such, their demographic information was reported as one combined group.

### Medical history

Data about the medical and family history and medication use, in particular on the presence of diabetes mellitus, hypertension and coronary disease, were collected through a structured interview, in combination with information provided by the study partner (if available), general practitioner and/or medical specialist.

### Ophthalmological examination

All participants underwent the following ophthalmological examinations: best corrected visual acuity, intra-ocular pressure, refraction data, slit lamp examination, indirect fundoscopy, fundus photography and OCT. Controls received tropicamide 0.5% to enable these examinations, nonagenarians both tropicamide 0.5% and phenylephrine 5% (as mydriasis was harder to achieve in these very aged patients). If a nonagenarian suffered from coronary stenosis, only tropicamide was given, due to the slight risk of phenylephrine inducing a coronary spasm. All photographs/OCT images were assessed by an experienced ophthalmologist (HTN or FDV) for unexpected pathology. Participants suffering from ophthalmological conditions severely interfering with the (neuro)retina or image quality were excluded from analyses (severe cataract, macular degeneration, glaucoma, diabetic retinopathy, vascular occlusions). Eyes with diseases considered to interfere with the OCT measurements excluded from OCT analyses could still be included in the SIVA analyses and vice versa (e.g. AMD with geographical atrophy interfered with OCT, but not fundus image analyses). This resulted in a slightly different study population for the OCT and SIVA analyses.

### Optical coherence tomography

Using spectral domain OCT (Spectralis, Heidelberg), dense macular scans (49 B-scans) and axonal ring scans around the optic nerve head (ONH) were acquired. Total retinal thickness and individual layer thickness was measured in the macular region. The following individual retinal layers were analyzed: retinal nerve fiber layer (RNFL), ganglion cell layer (GCL) and inner plexiform layer (IPL). A distinction was made between the inner and outer macular ring according to the standard Early Treatment and Diabetic Retinopathy Study (ETDRS) macular grid (1-3 mm around the fovea for inner ring and 3-6 mm around the fovea for outer ring). For further details on the acquiring of OCT data we refer to our earlier paper by van de Kreeke et al. [22].

### Fundus photography and quantitative assessment of retinal vasculature

Digital fundus images were made of the fundus of both eyes in all participants (Topcon TRC 50DX type IA). All images were graded by a trained grader (JAvdK) using the Singapore I Vessel Assessment (SIVA) software (version 3.0, National University of Singapore, Singapore) [9–11]. The following 7 retinal vascular parameters were analyzed: central retinal artery equivalent (CRAE), central retinal vein equivalent (CRVE), arteriole–venular ratio (AVR), fractal dimension of the arteriolar network (FDa), fractal dimension of the venular network (FDv), curvature tortuosity of the arterioles (cTORTa) and curvature tortuosity of the venules (cTORTv). All values for retinal vascular parameters were measured within zone C (i.e. 0.5–2 disc diameters around the optic nerve head). For further information on the analyses of fundus images we refer to our earlier paper by van de Kreeke et al. [23].

### Statistical analysis

First, we compared group means of all ocular outcome measures of the healthy and CI nonagenarian groups using linear regression, corrected for age, sex and diabetes. Additionally, mean differences between both nonagenarian groups and younger healthy controls were obtained using Generalized Estimating Equations (GEE). GEE was used to correct for clustering in the data from twin pairs in de control group. It also allowed us to correct for confounders such as sex and a diagnosis of diabetes. We deliberately did not correct this analysis for age, to illustrate the differences based on aging effects. Curvature tortuosity (cTORT) values for arteries and veins were log-transformed to normalize their distribution. All statistical analyses were performed using SPSS (IBM, version 22).

## Results

Table 1 shows the demographics of the combined study populations (for OCT and SIVA both) included for analyses.

**Table 1:** Demographics of the study population

	Controls	Healthy nonagenarians	CI nonagenarians
OCT analyses N	*172*	*52*	*23*
SIVA analyses N	*173*	*50*	*24*
Age (years)	*70.4 (±7.5)*	*92.4 (±1.9)*	*91.9 (±2.9)*
Sex, female N (%)	*105 (57.4%)*	*30 (52.6%)*	*20 (74.1%)*
BCVA (LogMAR)	*0.02 (±0.10)*	*0.14 (±0.21)*	*0.13 (±0.22)*
Intra-ocular pressure (mmHg)	*14.4 (±2.8)*	*15.9 (±2.0)*	*15.6 (±2.2)*
Spherical equivalent	*0.28 (±1.86)*	*−0.02 (±1.41)*	*0.05 (±0.13)*
MMSE (median, IQR)	*29.0 (29.0–30.0)*	*29.0 (28.0–30.0)*	*24.0 (20.0–26.0)*
Hypertension N (%)	*75 (41.0%)*	*23 (40.4%)*	*16 (59.3%)*
Diabetes mellitus N (%)	*8 (4.4%)*	*3 (5.3%)*	*2 (7.4%)*
Coronary disease N (%)	*18 (9.8%)*	*14 (24.6%)*	*7 (25.9%)*

Data are means from the groups combined unless otherwise specified

*CI* Cognitively Impaired, *N* Number of participants, *OCT* ptical Coherence Tomography, *SIVA* Singapore I Vessel Assessment, *BCVA* Best Corrected Visual Acuity, *MMSE* Mini-Mental State Exam, *IQR* Inter-Quartile Range, *CERAD* Consortium to Establish a Registry for Alzheimer’s Disease. Coronary disease in this case means a history of myocardial infarction or angina pectoris

When comparing the two nonagenarian groups, no significant differences in any of the ocular biomarkers were found (Table 2).

**Table 2:** Differences between means for ocular biomarkers in both the healthy and the CI nonagenarian groups

	Healthy nonagenarians	CI nonagenarians	SE	*p*-value
Macular retinal layer thickness:				
Total RT inner ring (μm)	*323.5*	*317.3*	*5.0*	*0.218*
Total RT outer ring (μm)	*282.2*	*276.1*	*4.0*	*0.133*
RNFL inner ring (μm)	*23.0*	*22.2*	*0.8*	*0.353*
RNFL outer ring (μm)	*36.4*	*35.5*	*1.4*	*0.518*
GCL inner ring (μm)	*43.9*	*42.9*	*1.6*	*0.550*
GCL outer ring (μm)	*30.0*	*29.9*	*1.1*	*0.940*
IPL inner ring (μm)	*36.6*	*35.5*	*1.0*	*0.275*
IPL outer ring (μm)	*25.5*	*24.9*	*0.8*	*0.449*
Peripapillary RNFL:				
Average (μm)	*88.3*	*89.1*	*2.7*	*0.762*
Nasal superior (μm)	*90.6*	*96.6*	*4.9*	*0.229*
Nasal (μm)	*67.1*	*69.9*	*3.5*	*0.428*
Nasal inferior (μm)	*101.9*	*102.3*	*5.3*	*0.940*
Temporal inferior (μm)	*123.0*	*122.1*	*5.2*	*0.868*
Temporal (μm)	*69.1*	*66.9*	*3.2*	*0.471*
Temporal superior (μm)	*118.9*	*118.0*	*4.5*	*0.854*
Retinal vascular parameters:				
CRAE	*123.6*	*120.3*	*2.3*	*0.156*
CRVE	*184.3*	*179.7*	*4.6*	*0.325*
AVR	*0.676*	*0.673*	*0.014*	*0.818*
FDa	*1.152*	*1.152*	*0.011*	*0.977*
FDv	*1.137*	*1.137*	*0.011*	*0.987*
cTORTa^a^	*−9.75*	*−9.80*	*0.062*	*0.407*
cTORTv^a^	*−9.73*	*−9.69*	*0.054*	*0.472*

Linear regression, corrected for age, sex and diabetes

*CI* Cognitively Impaired, *SE* Standard Error of difference, *RT* Retinal Thickness, *RNFL* Retinal Nerve Fiber Layer, *GCL* Ganglion Cell Layer, *IPL* Inner Plexiform Layer, *CRAE* Central Retinal Artery Equivalent, *CRVE* Central Retinal Vein Equivalent, *AVR* Arteriole-Venular Ratio, *FDa* Fractal Dimension of arteries, *FDv* Fractal Dimension of veins, *cTORTa* curvature Tortuosity of arteries, *cTORTv* curvature Tortuosity of veins

^a^Log transformation applied

**Fig. 1:**
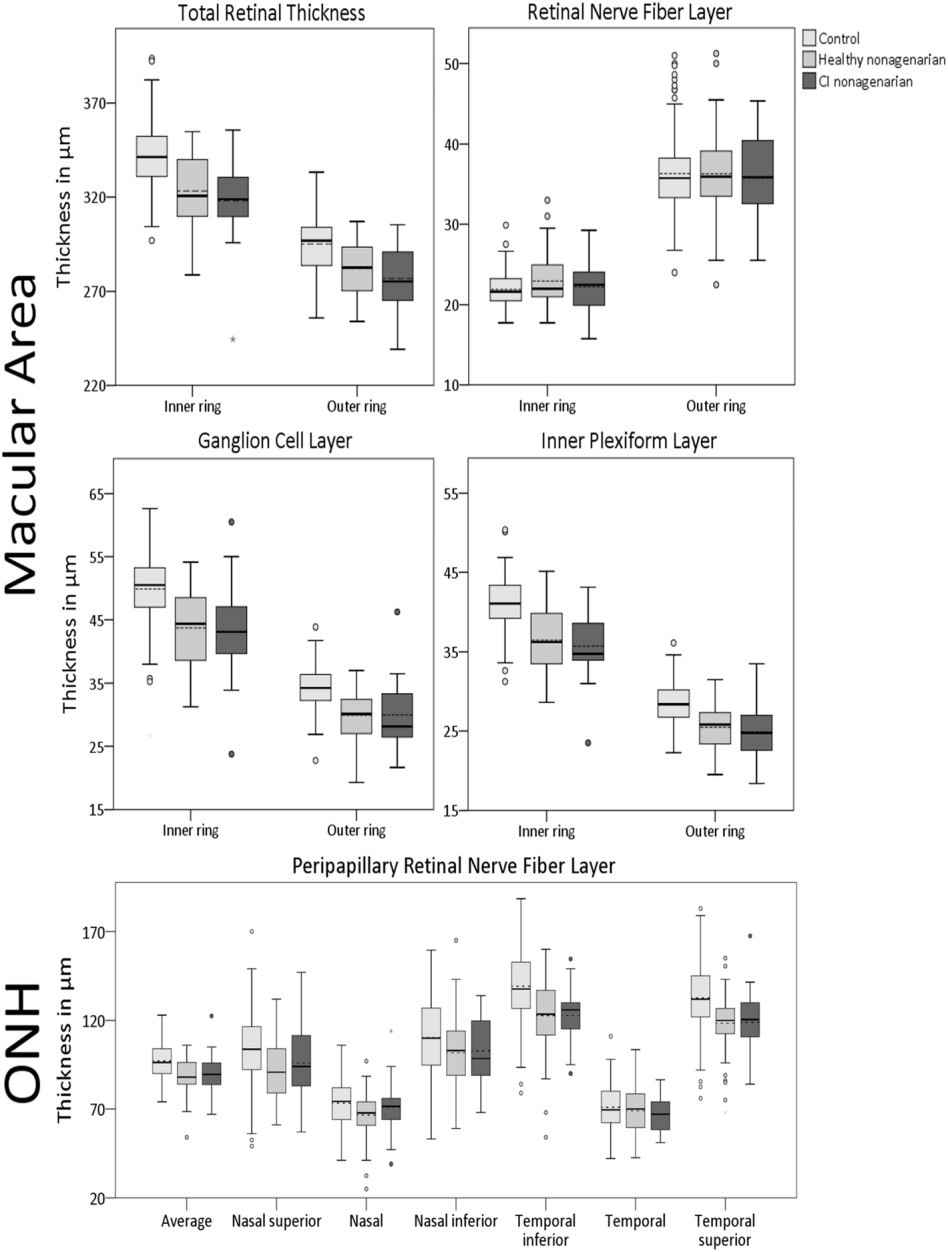
Boxplots for retinal layer thicknesses as measured with Optical Coherence Tomography in the 3 groups. Dotted line represents the mean. CI = Cognitively Impaired, ONH = Optic Nerve Head

**Fig. 2:**
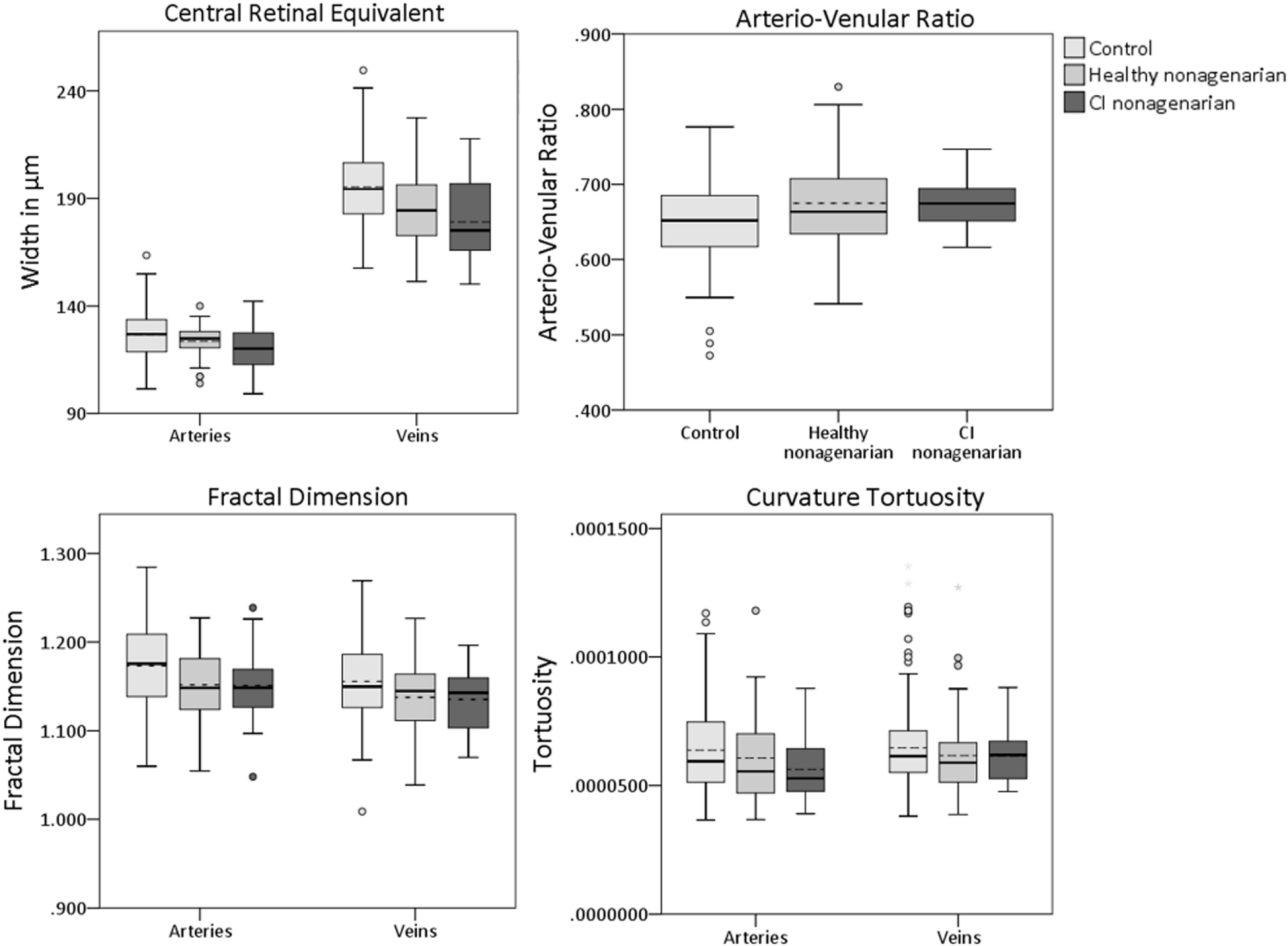
Boxplots for retinal vascular parameters obtained with Singapore I Vessel Assessment in the 3 groups. Dotted line represents the mean. CI = Cognitively Impaired, ONH = Optic Nerve Head

Both the healthy and the CI nonagenarians differed significantly in multiple ocular biomarkers when compared to the younger control group, with most ocular parameters being lower in the nonagenarian groups. 4 out of 22 biomarkers assessed differed significantly between healthy nonagenarians and younger controls, 1 out of 22 differed between CI nonagenarians and younger controls, and 14 out of 22 differed between both CI and healthy nonagenarians compared to younger controls. Figures 1 and 2 show boxplots for the 3 groups, Table 3 shows the mean differences of both nonagenarian groups compared to the control group, corrected for twin dependencies, sex and diabetes.

**Table 3:** Differences in ocular biomarkers of both nonagenarian groups compared to the younger control group

	Estimated marginal mean of controls	Mean difference compared to controls					
		Healthy nonagenarians	SE	p-value	CI nonagenarians	SE	*p*-value
Macular retinal layer thickness:							
Total RT inner ring (μm)	*340.1*	***−18.0***	***3.0***	***< 0.001***	***−22.5***	***5.0***	***< 0.001***
Total RT outer ring (μm)	*294.8*	***−12.7***	***2.4***	***< 0.001***	***−18.1***	***3.7***	***< 0.001***
RNFL inner ring (μm)	*21.9*	***1.0***	***0.5***	***0.032***	*0.4*	*0.7*	*0.559*
RNFL outer ring (μm)	*36.3*	*0.2*	*0.9*	*0.829*	*− 0.7*	*1.2*	*0.549*
GCL inner ring (μm)	*49.8*	***−6.1***	***0.9***	***< 0.001***	***− 6.5***	***1.7***	***< 0.001***
GCL outer ring (μm)	*34.2*	***−4.2***	***0.6***	***< 0.001***	***− 4.1***	***1.2***	***0.001***
IPL inner ring (μm)	*41.0*	***−4.5***	***0.6***	***< 0.001***	***−5.2***	***0.9***	***< 0.001***
IPL outer ring (μm)	*28.4*	***−2.9***	***0.4***	***< 0.001***	***−3.5***	***0.8***	***< 0.001***
Peripapillary RNFL:							
Average (μm)	*96.9*	***−8.6***	***1.6***	***< 0.001***	***−7.7***	***2.5***	***0.002***
Nasal superior (μm)	*103.7*	***−13.1***	***3.0***	***< 0.001***	*−7.1*	*4.9*	*0.145*
Nasal (μm)	*73.3*	***−6.5***	***2.3***	***0.004***	*−3.1*	*3.3*	*0.349*
Nasal inferior (μm)	*110.4*	***−8.7***	***3.7***	***0.018***	*−8.1*	*4.4*	*0.066*
Temporal inferior (μm)	*138.6*	***−15.4***	***3.3***	***< 0.001***	***−16.7***	***4.1***	***< 0.001***
Temporal (μm)	*70.7*	*−1.4*	*2.0*	*0.486*	*−4.1*	*2.5*	*0.108*
Temporal superior (μm)	*132.2*	***−13.5***	***2.9***	***< 0.001***	***−13.8***	***4.1***	***0.001***
Retinal vascular parameters:							
CRAE	*126.8*	***−3.1***	***1.5***	***0.038***	***−6.4***	***2.4***	***0.009***
CRVE	*195.3*	***−10.9***	***3.0***	***< 0.001***	***−14.4***	***4.2***	***0.001***
AVR	*0.652*	***0.024***	***0.010***	***0.013***	***0.017***	***0.009***	***0.045***
FDa	*1.173*	***−0.021***	***0.007***	***0.003***	***−0.021***	***0.010***	***0.035***
FDv	*1.156*	***−0.019***	***0.007***	***0.009***	***−0.018***	***0.009***	***0.040***
cTORTa^a^	*−9.690*	*− 0.057*	*0.041*	*0.171*	***−0.109***	***0.054***	***0.045***
cTORTv^a^	*−9.670*	*−0.054*	*0.038*	*0.153*	*−0.031*	*0.041*	*0.445*

Values in **bold** are significant at *p* < 0.05

*GEE* corrected for sex and a diabetes, *SE* Standard Error, *CI* Cognitively Impaired, *RT* Retinal Thickness, *RNFL* Retinal Nerve Fiber Layer, *GCL* Ganglion Cell Layer, *IPL* Inner Plexiform Layer, *CRAE* Central Retinal Artery Equivalent, *CRVE* Central Retinal Vein Equivalent, *AVR* Arteriole-Venular Ratio, *FDa* Fractal Dimension of arteries, *FDv* Fractal Dimension of veins, *cTORTa* curvature Tortuosity of arteries, *cTORTv* curvature Tortuosity of veins

^a^Log transformation applied

## Discussion

Ocular biomarkers showed no differences between cognitively healthy and cognitively impaired nonagenarians. However, nonagenarians did show extensive differences in their ocular biomarkers when compared to a younger control group, which were very similar to changes often attributed to AD in existing literature.

Our analyses showed no statistical difference in any of the studied ocular biomarkers between healthy and CI nonagenarians, suggesting that ocular biomarkers have a limited role in the detection of cognitive impairment in nonagenarians. A possible explanation for the lack of differences may be that other disorders that can have a profound effect on ocular biomarkers (such as hypertension, diabetes and coronary/cardiovascular disease), are also more prevalent in nonagenarians, and may obscure a possible neurodegenerative effect [24–26]. Another explanation can be that other aging related processes affect ocular biomarkers, again obscuring differences between the nonagenarian groups. Indeed, we found that both nonagenarian groups showed large differences in ocular biomarkers, including total/GCL/IPL thickness in the macula, average and most individual segments pRNFL thickness and all retinal vascular parameters except tortuosity (Figs. 1 and 2, Table 3), when compared to younger healthy controls. Several studies showed that aging is associated with thinning of the retina, as well as retinal vascular changes [15, 16, 26–30]. Normal aging unavoidably causes wearing of the body, as is seen in many other organ systems [31]. Especially in nonagenarians, compensatory mechanisms start failing, and such wear and tear effects become more and more pronounced, resulting in multimorbidity [32]. It is likely that this process also occurs in the retina. Blood vessels gradually start to decline due to aging effects like atherosclerosis, causing damage to (micro)vessels, leading to changes in retinal vascular parameters such as thinner vessel calibers and a lower fractal dimension of the vascular network [26]. This in turn may lead to a lower or insufficient blood and oxygen supply, causing damage to the neuronal tissue of the retina, resulting in the thinning of its layers [33, 34].

A problem we noticed when performing this study was the difficulty to make reliable images in nonagenarians. In this population there was a very high prevalence of ophthalmological pathology. Some of the diseases were already known at the time of the study and under treatment of an ophthalmologist, but several participants suffered, without their knowing, from ophthalmological pathology requiring medical attention. This resulted in a high exclusion of nonagenarian participants due to bilateral pathology, as illustrated in supplemental fig. 1. Approximately double the percentage of participants had to be excluded for OCT or SIVA analyses when comparing them with the younger control group. Additionally, acquiring good quality scans/images was harder in the nonagenarian groups, due to their lower endurance, physical impairments and, in the case of CI nonagenarians, cognitive functioning. This suggests that in a substantial (~ 25–30%) percentage of nonagenarians, ocular biomarkers for the diagnosis of dementia cannot be used, due to an inability to obtain reliable images/scans or bilateral ophthalmological pathology being present which interferes with the interpretation of the imaging.

A strength of the study was the extensive characterization of all participants. Participants were comprehensively screened for possible confounding pathologies, and were excluded (e.g. glaucoma, severe AMD, vascular occlusions etc.) or controlled for (diabetes).

Both nonagenarian groups were relatively small, limiting statistical power. Furthermore, as we included nonagenarians that were able to perform the study, which included 2–3 days of assessment (2 days in a hospital setting), this could also have introduced a bias, as only relatively healthy individuals of this older population will have been included in this study.

## Conclusion

We found no significant differences between the healthy and CI nonagenarian groups for any of the studied ocular biomarkers. We did find significant differences in 19 out of 22 of the ocular biomarkers assessed in this study when comparing either nonagenarian group to a healthy younger control grouper, suggesting a large effect of age on these biomarkers. The combination of these findings, along with the difficulties we encountered in obtaining suitable images in our nonagenarian population, leads to the conclusion that the use of ocular biomarkers in a population of the oldest-old is very limited.

## Supplementary information

**Additional file 1.** Reasons for excluding participants, categorized per group. Note that participants were only excluded if bilateral problems were present, explaining why the total N may be lower than the subdivided numbers taken together (i.e. 1 eye of a participant may fall in 1 category, and the contralateral eye in another). CI = Cognitively Impaired, OCT = Optical Coherence Tomography, SIVA = Singapore I Vessel Assessment, AMD = Age-related Macular Degeneration, ERM = Epiretinal Membrane, PPA = Peripapillary Atrophy, CSC = Central Serous Chorioretinopathy.

## Data Availability

The datasets used and/or analyzed during the current study are available from the corresponding author on reasonable request.

## Abbreviations

AD: Alzheimer’s Disease
aMCI: amnestic Mild Cognitive Impairment
AMD: Age-related Macular Degeneration
AVR: Arteriole-Venular Ratio
BCVA: Best Corrected Visual Acuity
CDR: Clinical Dementia Rating
CERAD: Consortium to Establish a Registry for Alzheimer’s Disease
CI: Cognitively Impaired
CRAE: Central Retinal Artery Equivalent
CRVE: Central Retinal Vein Equivalent
cTORTa: Curvature Tortuosity of arteries
cTORTv: Curvature Tortuosity of veins
ETDRS: Early Treatment and Diabetic Retinopathy Study
FDa: Fractal Dimension of arteries
FDv: Fractal Dimension of veins
GCL: Ganglion Cell Layer
GDS: Geriatric Depression Scale
GEE: Generalized Estimating Equations
IPL: Inner Plexiform Layer
MMSE: Mini Mental State Exam
OCT: Optical Coherence Tomography
ONH: Optic Nerve Head
pRNFL: peripapillary Retinal Nerve Fiber Layer
RNFL: Retinal Nerve Fiber Layer
RT: Retinal Thickness
SIVA: Singapore I Vessel Assessment
TICS-m: Telephone Interview for Cognitive Status modified
