# Supplementary material for: Ocular biomarkers for cognitive impairment in nonagenarians; a prospective cross-sectional study
Source: BMC Geriatr. 2020 Apr 28;20:155. doi: 10.1186/s12877-020-01556-1 (PMC7189586; doi:10.1186/s12877-020-01556-1)
Supplement: Supplementary file 1 — Additional file 1. Reasons for excluding participants, categorized per group. Note that participants were only excluded if bilateral problems were present, explaining why the total N may be lower than the subdivided numbers taken together (i.e. 1 eye of a participant may fall in 1 category, and the contralateral eye in another). CI = Cognitively Impaired, OCT = Optical Coherence Tomography, SIVA = Singapore I Vessel Assessment, AMD = Age-related Macular Degeneration, ERM = Epiretinal Membrane, PPA = Peripapillary Atrophy, CSC = Central Serous Chorioretinopathy. [file 12877_2020_1556_MOESM1_ESM.pdf]

Additional file 1: Reasons for excluding participants, categorized per group.

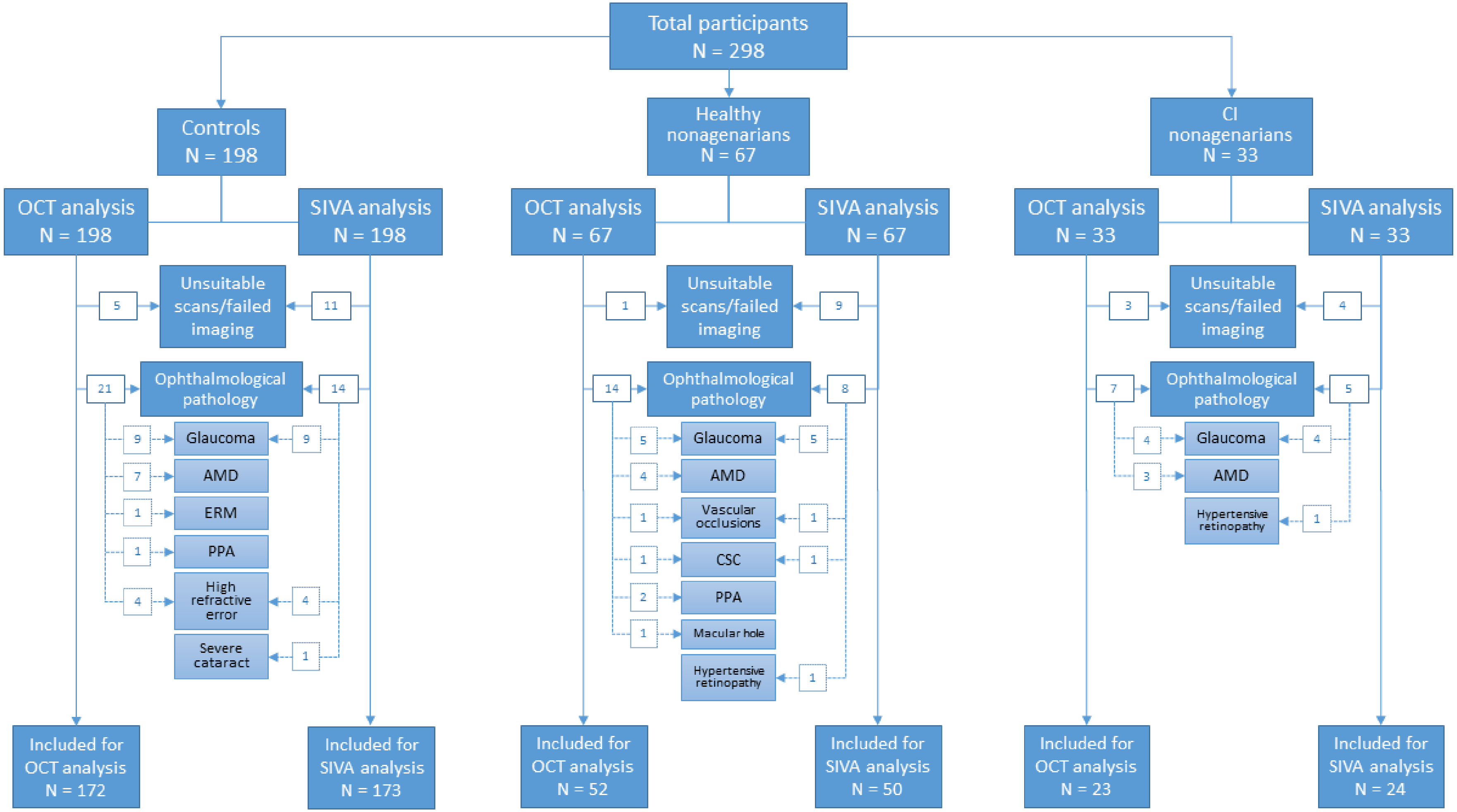

Note that participants were only excluded if bilateral problems were present, explaining why the total N may be lower than the subdivided numbers taken together (i.e. 1 eye of a participant may fall in 1 category, and the contralateral eye in another). CI = Cognitively Impaired, OCT = Optical Coherence Tomography, SIVA = Singapore I Vessel Assessment, AMD = Age-related Macular Degeneration, ERM = Epiretinal Membrane, PPA = Peripapillary Atrophy, CSC = Central Serous Chorioretinopathy.
